# Comparative Study of Antioxidant and Pro-Oxidant Properties of Homoleptic and Heteroleptic Copper Complexes with Amino Acids, Dipeptides and 1,10-Phenanthroline: The Quest for Antitumor Compounds

**DOI:** 10.3390/molecules26216520

**Published:** 2021-10-28

**Authors:** Nicolás Veiga, Natalia Alvarez, Eduardo E. Castellano, Javier Ellena, Gianella Facchin, María H. Torre

**Affiliations:** 1 Química Inorgánica, DEC, Facultad de Química, Universidad de la República, Av. Gral. Flores 2124, Montevideo C.P. 11800, Uruguay; nveiga@fq.edu.uy (N.V.); nalvarez@fq.edu.uy (N.A.); 2 Laboratório Multiusuário de Cristalografia Estrutural, Instituto de Física de São Carlos, Universidade de São Paulo, São Carlos CEP 13566-590, Brazil; pino@ifsc.usp.br (E.E.C.); javiere@if.sc.usp.br (J.E.)

**Keywords:** antioxidant, pro-oxidant, copper complexes, SOD-like activity, TBARS, X-ray diffraction, DFT analysis

## Abstract

In a search for new antitumoral agents, a series of homoleptic copper(II) complexes with amino acids and dipeptides, as well as heteroleptic complexes containing both dipeptides and 1,10-phenanthroline, were studied. Furthermore, a single-crystal structure containing alanyl-leucinato ([Cu_3_(AlaLeu)_3_(H_2_O)_3_(CO_3_)]·PF_6_·H_2_O), which is the first homotrinuclear carbonato-bridged copper(II) complex with a dipeptide moiety, is presented. To assess possible antitumor action mechanisms, we focused on the comparative analysis of pro- and antioxidant behaviors. Pro-oxidant activity, in which the reactive oxygen species (ROS) formed by the reaction of the complexes with H_2_O_2_ produce oxidative damage to 2-deoxy-d-ribose, was evaluated using the TBARS method. Additionally, the antioxidant action was quantified through the superoxide dismutase (SOD)-like activity, using a protocol based on the inhibitory effect of SOD on the reduction of nitrobluetetrazolium (NBT) by the superoxide anion generated by the xanthine/xanthine oxidase system. Our findings show that Cu–amino acid complexes are strong ROS producers and moderate SOD mimics. Conversely, Cu–dipeptide–phen complexes are good SOD mimics but poor ROS producers. The activity of Cu–dipeptide complexes was strongly dependent on the dipeptide. A DFT computational analysis revealed that complexes with high SOD-like activity tend to display a large dipole moment and condensed-to-copper charge, softness and LUMO contribution. Moreover, good ROS producers have higher global hardness and copper electrophilicity, lower copper softness and flexible and freely accessible coordination polyhedra.

## 1. Introduction

Metal-based drugs have played an important role in cancer treatment and in the expansion of research groups focused on the development of new antitumor drugs. It is well-known that cisplatin and subsequent generations (carboplatin, oxalylplatin, heptaplatin and picoplatin) have shown high effectiveness for the treatment of tumors in clinical practice but also present several toxic effects [1]. In spite of these successful clinical contributions, the development of new potential anticancer metallopharmaceuticals remains mainly academic. Kellett et al. [2] suggest that this could be due to the fact that metal-coordination compounds are reactive. They can exchange ligands, participate in redox reactions and have multiple mechanisms of action. This intrinsic reactivity of the coordination compounds presents two opposing aspects that should be evaluated. On one hand, it contributes to the prodrug character of these compounds, whereas, on the other hand, it has established the idea that these compounds’ mechanisms of action are difficult to study and understand. Fortunately, the latter is changing and several metal-based drugs are in clinical trials and many more are awaiting ethical approval to join the tests [3,4,5].

In the search for new drugs with lower toxic effects, several research groups have focused their work on complexes with endogenous metals; for instance, copper, the coordination compounds of which were found to be promising antitumor agents by Krasnovskaya et al. [6]. Antitumor copper complexes can act following different mechanisms of action. Some of them involve the interaction of the copper complex with critical biomolecules in tumor cell development, carrying out Lewis acid–base reactions in a general sense. Some examples include the interaction with nucleic acids and their constituents [7]; the interaction with crucial proteins, such as topoisomerases, kinases and proteasomes [8]; and the interaction with angiogenesis inhibitors [9]. However, their therapeutic efficacy as antitumor agents is not limited to these actions. 

Due to the redox activity of copper complexes, they can present a dual role. They can act as protective antioxidants destroying, for example, free radicals. For this purpose, the human organism has several endogenous enzymatic systems to deal with the oxidative stress. The superoxide dismutases are one set of detoxifying systems, which, as their name implies, catalyze the superoxide radical dismutation. In this regard, several copper complexes have shown evidence that they play a protective role in oxidative processes, acting as good mimics of the Cu/Zn SOD (SOD1) [10,11,12,13]. Copper complexes are also able to act as pro-oxidants, producing free radicals that cause oxidative damage to different substrates, including proteins, nucleic acids, lipids [6,14,15] and toxic molecules [16], and inducing apoptosis in tumor cells [4]. It has been well-established that some complexes are capable of generating reactive species in measurable amounts, particularly in the presence of hydrogen peroxide [17,18,19]. In this scenario, some copper complexes combine both antioxidant and pro-oxidant modes of action. It is well-known that the disturbance of the redox balance between free radical pro-oxidants and antioxidant systems can result in oxidative stress, which is related to different diseases like cancer. The compounds with both antioxidant and pro-oxidant activities could act as protective molecules that dismutate superoxide radicals, as well as functioning as antitumor species since they can produce ROS, which can damage different biomolecules and induce cellular senescence and death [20]. One example of compounds presenting both activities is the case of cytotoxic Cu(II)–diimine compounds, which can efficiently dismutate the superoxide radical anion, generating at the same time a noteworthy amount of ROS in the presence of hydrogen peroxide [21]. Similar dual behavior was reported by Pires dos Santos et al., who worked with di-Schiff base copper(II) complexes [22], and by Simunkova et al., who studied copper(II) complexes with non-steroidal anti-inflammatory drugs [23].

Another aspect related to the redox behavior is the ability of copper to transport coordinated active molecules inside the cell, where they are released upon Cu(II) bioreduction before they can act [24].

In recent decades, our group has synthesized and characterized several copper complexes with amino acids, dipeptides and 1,10-phenanthroline [25,26,27,28,29,30]. In most cases, adequate single crystals were obtained and their crystal structures determined by X-ray diffraction. Nevertheless, up to now, the crystallization of the homoleptic copper(II)–dipeptide complex with alanyl-leucinato (AlaLeu) has been elusive. In fact, there is only one report of a prepared Cu–AlaLeu complex, in which no crystal structure is available [31]. This is particularly intriguing, since there is extensive work in this field. Indeed, the first copper(II)–dipeptide crystal structure dates back to 1961, with the Cu–GlyGly structure reported by Strandberg et al. [32], and the most recent corresponds to Cu–GlyThr, reported in 2017 by Ma et al. [33].

In this article, the crystal structure for the copper(II)–AlaLeu complex is presented for the first time. Interestingly, it constitutes the first homotrinuclear carbonato-bridged copper(II) complex bearing a dipeptide ligand. Furthermore, and following the research on promising antitumor compounds, the antioxidant behavior of a series of copper complexes with amino acids, dipeptides and 1,10-phenanthroline was compared by measuring the SOD-like activity. To learn more about their reactivity, evaluation of the pro-oxidant activity by means of an assessment of the oxidative damage to 2-deoxy-d-ribose (using the TBARS method) was performed. Even though there are some records of the antioxidant and pro-oxidant activities of the selected copper(II) complexes, the information has never been methodically analyzed. To this end, a comparative study of the experimental evidence collected by our group and others was carried out, with special emphasis on the relationship between the activities and the available structural information. In order to shed light on the structural and electronic basis behind the redox behavior, a DFT computational analysis was performed on selected systems.

## 2. Results and Discussion

### 2.1. Synthesis

The copper complexes included in this article were selected on the basis that they are chemically related but present different copper environments in solution, and some of them exhibit cytotoxic activities. They were synthesized with high purity and good yields. The general stoichiometries were [Cu(AA_−1_)_2_] nH_2_O, [Cu(dipH_−2_)]⋅nH_2_O and heteroleptic [Cu(dipH_−2_)phen]⋅nH_2_O, where AA = amino acid, dipH = L-dipeptide and phen = 1,10-phenanthroline. The charge neutrality in the complexes was achieved by the monodeprotonation of each AA and bideprotonation of each dipeptide, both in the homoleptic and the heteroleptic complexes.

The code used was [Cu(Gly)_2_]·H_2_O (Cu–Gly), [Cu(Ala)_2_] (Cu–Ala), [Cu(Val)_2_] (Cu–Val), [Cu(Ser)_2_] (Cu–Ser), [Cu(Ile)_2_]·H_2_O (Cu–Ile), [Cu(GlyVal)]·1/2H_2_O (Cu–GlyVal), [Cu(ValGly)] (Cu–ValGly), [Cu(AlaGly)] (Cu–AlaGly), [Cu(AlaPhe)]·1/2H_2_O (Cu–AlaPhe), [Cu(PheAla)]·1/2H_2_O (Cu–PheAla), [Cu(AlaLeu)_3_(H_2_O)(CO_3_)]·PF_6_·H_2_O (Cu–AlaLeu), [Cu(AlaGly)(phen)] (Cu–AlaGly–phen), [Cu(PheAla)(phen)] (Cu–PheAla–phen), [Cu(PheVal)(phen)] (Cu–PheVal–phen). 

Almost all the homoleptic copper dipeptide complexes presented similar stoichiometry, except for [Cu(AlaLeu)_3_(H_2_O)(CO_3_)]·PF_6_·H_2_O where the carbonate anion acts as a bridging ligand, which was not observed for the other complexes synthesized and crystallized following the same procedure.

### 2.2. Crystal Structure of Cu–AlaLeu

The obtained crystal structure constitutes the first copper(II) complex containing alanyl-leucinato and also the first homotrinuclear carbonato-bridged copper(II) complex with a dipeptide moiety: μ_3_-carbonato-tris(alanyl-leucinato)-tris(aquo)-tris-copper(II) hexafluorophosphate monohydrate. The obtainment of this particular structure can be considered as a serendipitous event, since it was obtained with the same methodology used for the rest of the Cu(II)–dipeptide complexes previously reported by our group.

A structural search for copper complexes that contained carbonate anion acting as a bridging ligand between at least three copper centers was conducted using Conquest in the Cambridge Structural Database (CSD) v5.42 with the Feb21 update [34]. The search yielded a total of 71 crystal structures, where 35 corresponded to homo- and hetero-polynuclear complexes with more than three copper(II) centers, as well as 1- and 3D infinite arrangements. The remaining 36 corresponded to discrete carbonato-bridged trinuclear copper(II) complexes. The most common counterions included ClO_4_^−^ (54%), PF_6_^−^ (11%), F_3_CSO_3_^−^ (9%), BF_4_^−^ (9%) and NO_3_^−^ (6%). A complete list of CSD Refcodes and counterions is available in Table S1 in the Supplementary Materials.

**Figure 1:**
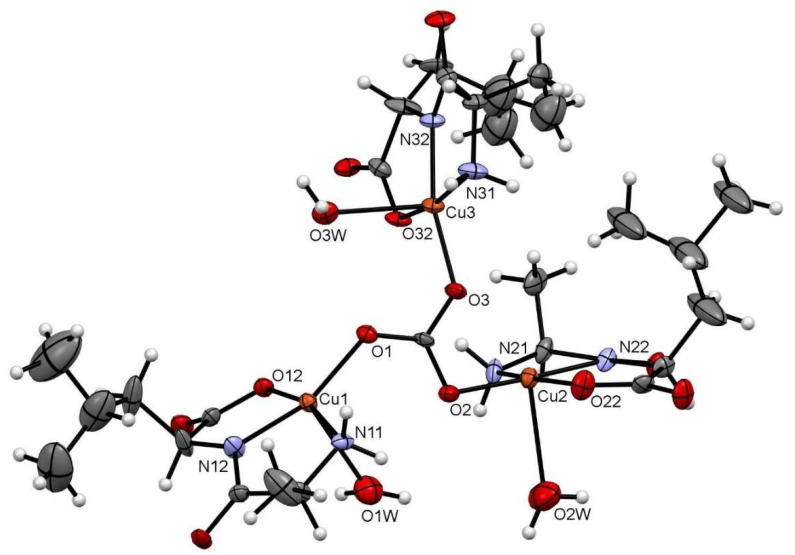
ORTEP-type diagram of the asymmetric unit of Cu–AlaLeu with 50% probability ellipsoids. The hexafluorophosphate anion and the hydration water molecule are omitted for clarity. The atom numbering scheme for coordinating atoms is included. Atom color code: C (gray), O (red), N (blue), Cu (orange) and H (white).

Figure 1 depicts the asymmetric unit content of Cu–AlaLeu; the hexafluorophosphate anion and the hydration water molecule are omitted for clarity. In all cases the dipeptide acts as a tridentate ligand, coordinating through the amino terminal N and O atoms and amidic N atom, similarly to previously reported structures. The copper center sits on a nearly perfect elongated square pyramidal geometry, as evaluated through the τ factor, where τ = 0 for a perfect square-based pyramid and 1 for a trigonal bipyramid [35]. For the three metal centers in the structure, the τ values are 0.04, 0.19 and 0.02 for Cu1, Cu2 and Cu3, respectively. The dipeptide and an O atom from the carbonate bridge comprise the equatorial plane; meanwhile, a water molecule completes the axial position. This can also be confirmed by the bonding distances in the coordination sphere shown in Table 1, where the CuX–OXW bond distances are longer than the rest of the coordinative bonds. The observed arrangement with the dipeptide ligand in the equatorial plane is the same as the one previously published for the complexes Cu–AlaVal, Cu–AlaPhe [36], Cu–AlaIle, Cu–AlaThr and Cu–AlaTyr [30].

**Figure 2:**
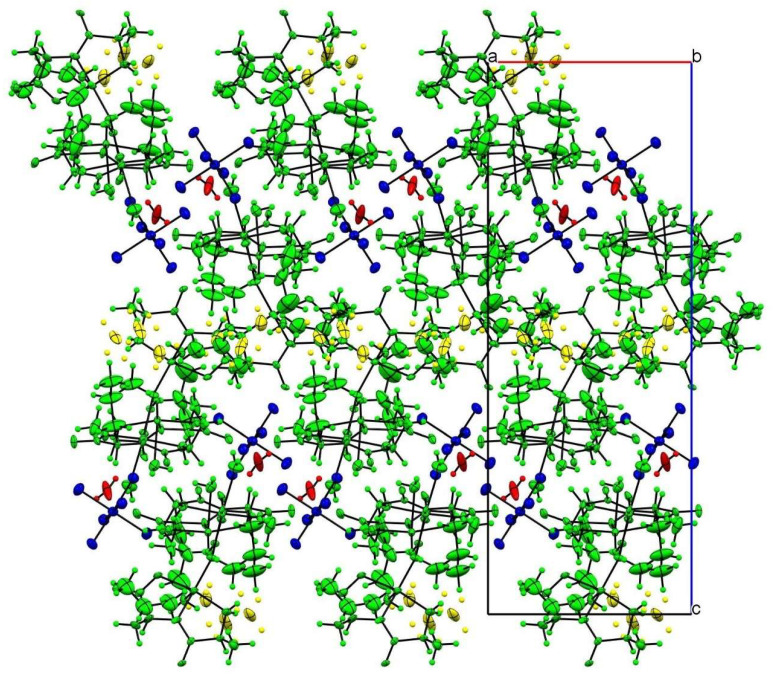
Crystal-structure packing view along the *b* axis. Color code follows the symmetry equivalence: complex cations (green), disordered alkyl groups (yellow), hexafluorophosphate (blue) and water hydration molecule (red).

In the crystal packing, the most important intermolecular interactions are of an electrostatic nature, given the cationic nature of the trinuclear copper(II) complex. As can be seen in Figure 2, the charge is balanced by the hexafluorophosphate anion, which occupies the larger voids in the structure following a zigzag arrangement. 

### 2.3. Structural Characterization in Solution

**Figure 3:**
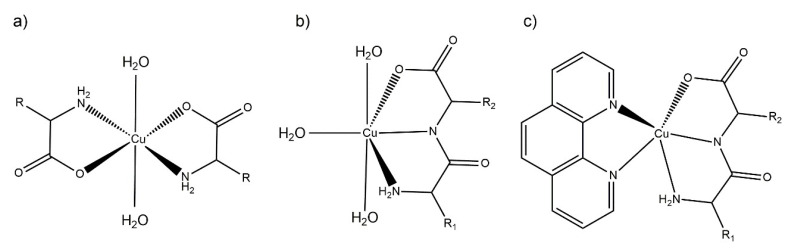
Scheme representation of the proposed aqueous solution coordination environment for: (**a**) Cu–AA, (**b**) Cu–dipeptide and (**c**) Cu–dipeptide–phen complexes. R_1_ and R_2_ represent different substituents.

The antioxidant and pro-oxidant activity tests were carried out in aqueous solution (see below). Therefore, to aid in the interpretation of the results, the structural parameters in solution and their evolution over time was determined. The proposed aqueous solution structures are depicted in Figure 3, based on the structural information derived from the electronic spectra and the results from the solid state characterization, mainly single-crystal X-ray diffraction.

The λ and molar absorptivities of absorption maxima for each spectrum in aqueous solution are presented in Section 3.2. The main absorption band in the visible spectra was assigned to *d-d* transitions. The complexes maintained similar visible spectra in aqueous solution for at least 30 days, accounting for their stability in aqueous solution. According to the electronic spectra, and taking into account the previous structural information in the solid state, the equatorial environment could be determined using the Prenesti equations [37,38]. In the Cu(II)–AA complexes, the theoretical wavelength considering the Prenesti equations with an equatorial environment formed by two nitrogen atoms from the terminal amine (N_a_) and two carboxylate oxygen (O_c_) atoms (N_a_N_a_O_c_O_c_) was 623 nm. The experimental values (612–632 nm) were well within the expected range, indicating that the main species in solution would be the one shown in Figure 3a.

For Cu(II)–dipeptide complexes, the theoretical wavelength considering the Prenesti equations with an equatorial environment formed by two nitrogen atoms from the amine (N_a_) and amide (N_p_) residues, one oxygen atom from the carboxylate group (O_c_) and one from a water (O_w_) molecule (N_a_N_p_O_c_O_w_) was 627 nm. The experimental results (625–636 nm) agreed with the modeling in which the dipeptide remained as tridentate ligand in the equatorial plane in solution, as observed in the solid state (Figure 3b).

The λ_max_ values of Cu(II)–dipeptide–phen complexes were reported previously by Iglesias et al. [26]. The spectra present the characteristic shoulder of pentacoordinated copper environments, and the Prenesti prediction agrees with the proposed N_3_O chromophore based on the solid-state information (Figure 3c).

### 2.4. Assessment of Oxidative Damage to 2-Deoxi-d-ribose (Using TBARS Method)

**Figure 4:**
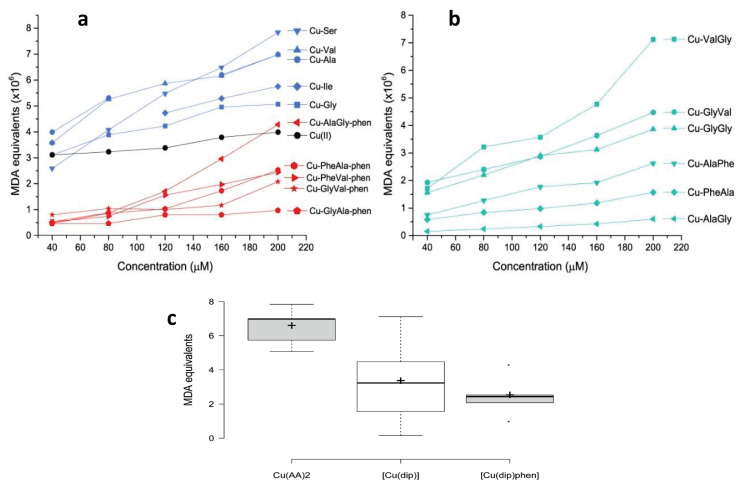
Oxidative damage to 2-deoxy-d-ribose induced by Cu(II) complexes expressed as MDA equivalents for: (**a**) copper complexes with amino acids and with dipeptide–phenantroline and (**b**) copper complexes with dipeptide. In (**c**), comparative boxplots of the MDA equivalents (×10^6^) at 200 μM are shown for each group of complexes.

The pro-oxidant activity of the selected copper complexes was assessed by the reaction with 2-deoxi-d-ribose and using a TBARS protocol. This methodology can be used to determine the ability of the complexes to generate OH from H_2_O_2_, which subsequently oxidizes 2-deoxy-d-ribose into malondialdehyde (MDA). MDA then reacts with 2-thiobarbituric acid (TBA), yielding a colored species the concentration of which can be followed through its electronic spectra and expressed as MDA equivalents [15,18]. The results are shown in Figure 4.

As observed in Figure 4a, even though all the complexes produced OH from H_2_O_2_, including free Cu(II), the homoleptic complexes with amino acids were the most active. In fact, the average MDA equivalent was higher for Cu–AA complexes than for the rest of the tested compounds. This difference was statistically significant according to the Mann–Whitney *U* exact test: *p* = 0.05 for Cu–AA/Cu–dipeptide and *p* = 0.008 for Cu–AA/Cu–dipeptide–phen pairs. Interestingly, some of the Cu–AA complexes displayed antiproliferative activity against tumor cell lines (SNU484 and SNU638), and a part of this effect could have been due to the high production of free radicals [39]. From a structural point of view, in Cu–AA complexes the metal center is coordinated to two amino acids in the equatorial plane, leaving two coordination sites that are occupied by water molecules. These two labile positions are expected to be significantly reactive, binding radical precursor substrates and giving rise to a high level of ROS production.

Conversely, the heteroleptic Cu–dipeptide–phen complexes were less active, displaying lower MDA equivalents than the Cu–AA compounds (Figure 4c).

It has also been found that they present high antiproliferative activity [26]. It is possible that Cu–dipeptide–phen complexes exert their action through other mechanisms apart from ROS species production.

For these heteroleptic complexes, a pentacoordinated geometry is preferred, making the copper(II) center less accessible for the substrate, which could be the cause of this weaker ability to produce OH.

According to Figure 4b, the pro-oxidant activity of the Cu–dipeptide complexes has substantial variability. There are compounds for which the activity is lower and similar to that of the Cu–dipeptide–phen complexes (Cu–AlaGly, Cu–AlaPhe, Cu–PheAla), while others present higher values closer to those found for Cu–AA. The two least active dipeptide complexes, Cu–AlaPhe and Cu–PheAla, were previously biologically evaluated and showed good antiproliferative activity against breast cancer cells and low toxicity for fibroblasts [25]. This result suggests that the ROS production is not the only chemical process inducing the cytotoxicity.

In conclusion, our results show that homoleptic complexes with amino acids display stronger pro-oxidant activities than those of free copper(II), while most of the Cu–dipeptide and Cu–dipeptide–phen complexes were poorer OH producers (Figure 4a,b). In this regard, it is evident that the structural and electronic features of the complexes, including the copper coordination geometry and donor sets, modulated the pro-oxidant activity. This was also observed by Carvalho do Lago et al., who compared copper(II)–imine complexes (stabilized by π-interaction) and copper(II)–peptide ones [17].

### 2.5. Determination of SOD-like Activity

The antioxidant behavior of the selected complexes was comparatively evaluated by determining the SOD-like activity. This parameter was measured by employing Beauchamp and Fridovich’s protocol as improved by Imanari et al. [40,41]. It is based on the inhibitory effect of SOD on the reduction of nitrobluetetrazolium (NBT) by the superoxide anion generated by the xanthine/xanthine oxidase system. The results are expressed as IC_50_, i.e., the concentration required to yield 50% inhibition of the NBT reduction. The obtained values are listed in Table 2, along with previously reported results.

In general terms, all the compounds showed SOD-like activity, including free Cu(II). As expected, all the tested complexes presented lower SOD-like activity than that of the native SOD, a highly evolved enzyme that can efficiently detoxify organisms from the O_2_^−^ radical. According to Roberts and Robinson’s classification the active complexes are those that display IC_50_ lower than 20 µM [42]. Below that value, the copper complexes usually present antitumoral or anti-inflammatory activities associated with the dismutation of the superoxide radical. This was the case for the heteroleptic Cu–dipeptide–phen complexes, for which the IC_50_ values lay close to 5 μM (standard deviation = 4.0 μM). They were particularly efficient at mimicking SOD, having average IC_50_ values that were lower than those for the rest of the homoleptic complexes. This difference was, in fact, statistically significant (Mann–Whitney *U* exact test: *p* = 0.04 for Cu–AA/Cu–dipeptide–phen and *p* = 0.05 for Cu–dipeptide/Cu–dipeptide–phen pairs).

As Table 2 shows, the Cu–AA complexes are moderate SOD mimics, with IC_50_ values localized around 33 μM with really low dispersion (standard deviation = 2.5 μM). Interestingly, most of them showed high pro-oxidant activity, as discussed in Section 2.4. Possibly, both redox mechanisms, antioxidant and pro-oxidant, are operative when they act as antiproliferative complexes.

As was observed for the pro-oxidant behavior, Cu(II)–dipeptide complexes show very variable antioxidant ability, with IC_50_ values ranging between 5 and 124 μM (Table 2; standard deviation = 40.9 μM). Clearly, the structural and electronic characteristics of the dipeptides exert a profound effect on their redox reactivity. Within this group, Cu–AlaPhe was substantially more active than the other complexes (IC_50_ = 5.0 μM), exhibiting similar in vitro antiproliferative activity as Cu–PheAla [22]. On the other hand, Cu–AlaGly presented the lowest SOD activity and it was also a poor pro-oxidant compound at low concentrations (Figure 4b), in line with the low reported antiproliferative activity [43]. 

### 2.6. Structural and Electronic Determinants of the Redox Behavior

The experimental findings described so far point to the conclusion that the structural and electronic features of the complexes brought about by the different ligands exert a profound effect on the antioxidant and pro-oxidant activities. Similar conclusions have been reached by several authors [11,47,48]. To gain insight into this phenomenon, a DFT computational analysis was performed on a selection of five complexes: Cu–Ala, Cu–AlaPhe, Cu–AlaGly, Cu–PheAla and Cu–AlaGly–phen. The first complex was included since it is one of the most active Cu–AA complexes in producing OH at a wide range of concentrations (Figure 4a). The second and third complexes were chosen because they represent extremes in the antioxidant activity of the dipeptides’ complexes (see Table 2). The Cu–PheAla complex was added to the group since it exhibits lower SOD-like activity than Cu–AlaPhe, even though both species are isomers. Lastly, Cu–AlaGly–phen was included to represent the heteroleptic phen complexes because its antioxidant activity (IC_50_ = 10 μM) is significantly higher than its homoleptic counterpart Cu–AlaGly (IC_50_ = 124 μM; Table 2).

**Figure 5:**
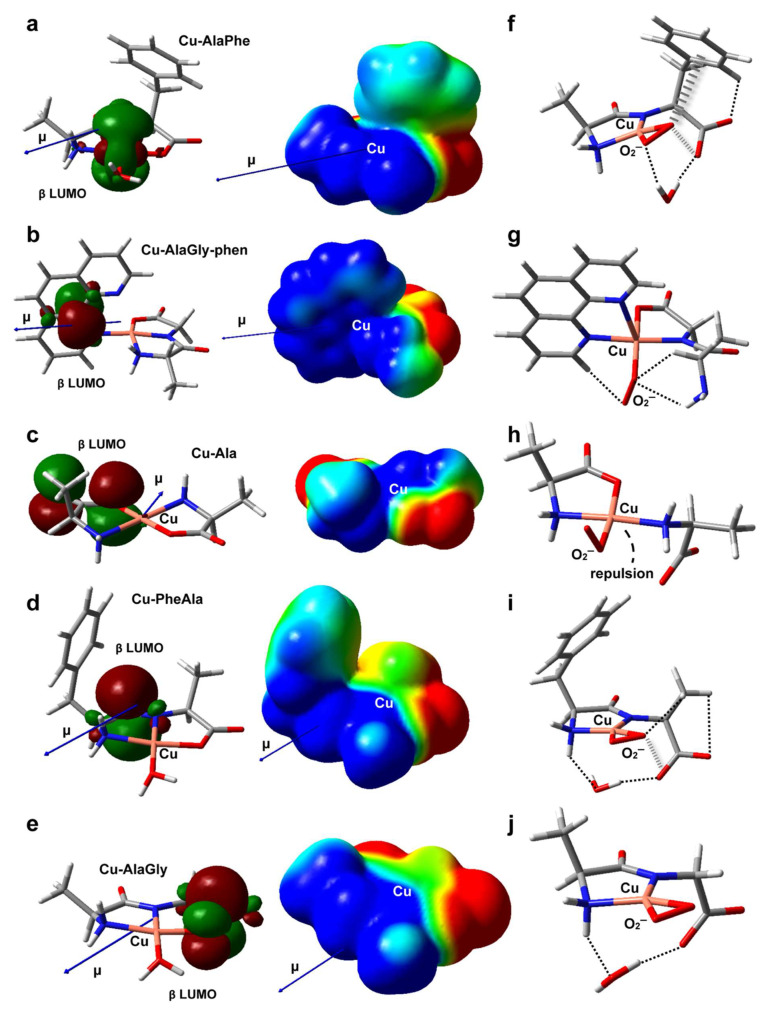
DFT-optimized geometries in aqueous solution of Cu–AlaPhe) (**a**), Cu–AlaGly)–phen (**b**), Cu–Ala (**c**), Cu–PheAla) (**d**) and Cu–AlaGly (**e**). The electric dipole moment vector (μ), the β LUMO spatial distribution and the electrostatic potential mapped onto an isodensity surface (isodensity value = 0.001 e; scale = −50 (red) to +50 (blue) mV) are also shown. In (**f**–**j**), the optimized structures of the corresponding superoxide complexes are depicted, with the noncovalent intramolecular interactions represented as dashed (H-bonds) or hatched (anion-π interactions) lines. Atom color code: C (gray), H (white), N (blue), O (red), Cu (pink).

**Figure 6:**
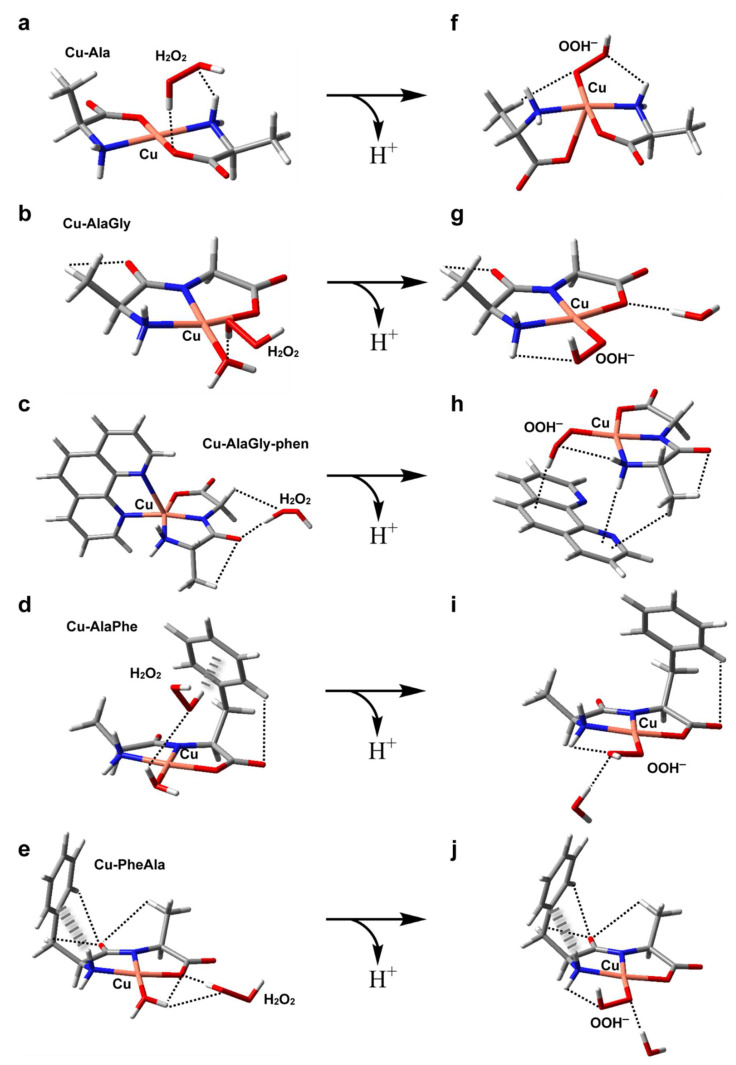
DFT-optimized geometries in aqueous solution of the adducts formed by hydrogen peroxide with Cu–Ala (**a**), Cu–AlaGly (**b**), Cu–AlaGly–phen (**c**), Cu–AlaPhe (**d**) and Cu–PheAla) (**e**). In (**f**–**j**), the optimized structures of the corresponding OOH^−^ complexes are depicted, with the noncovalent intramolecular interactions represented as dashed (H-bonds) or hatched (H-π interactions) lines. Atom color code: C (gray), H (white), N (blue), O (red), Cu (pink).

The structures of the five metal complexes were optimized in solution at the B3LYP/6-31 + G(d,p) level of theory (see the Materials and Methods section for further details). Starting from the DFT-optimum geometries, some meaningful reactivity indexes were determined following *conceptual density functional theory* (Table 3 and Table 4), including global and condensed-to-copper descriptors [49]. To complement the analysis, Figure 5a–e depict the structures, electric dipole moment vector (μ) and the electrostatic potential (ESP) for each complex, along with the spatial distribution of the β LUMO. Given the fact that the stability of the intermediates may also have an impact on the redox reactivity, the adducts formed between the copper complexes and O_2_^−^, H_2_O_2_ or OOH^−^ (the deprotonated form of hydrogen peroxide) were modeled as well (see Figure 5f–j and Figure 6a–j). The weak intramolecular interactions established in those species were identified using the noncovalent interaction method (NCI) (Figures S1 and S2) [50].

#### 2.6.1. Antioxidant Activity

The experimental results revealed that the heteroleptic Cu–dipeptide–phen complexes were very efficient at mimicking SOD, giving rise to a high level of O_2_^−^ dismutation. According to Table 3, the Cu–AlaGly–phen complex had the largest dipole moment and the highest atomic charge at the metal center, possibly due to the π-acceptor ability of phen [51]. This suggests that, for this group of complexes, the high reactivity towards the superoxide anion is promoted mainly by electrostatic interactions that stabilize the transition states, lowering the activation barriers. Indeed, the inclusion of the coligand phen triggers a rearrangement of the electrostatic potential, giving rise to a cone-shaped positively charged zone around the copper ion that tunnels the O_2_^−^ anion along the direction of the dipole moment vector (μ) towards the metal center (compare Figure 5b,e). Interestingly, the phen moiety also plays a role in the stabilization of the O_2_^−^ ligand within the complex (Figure 5g) through the formation of a nonconventional C-H/O H-bond. This type of interaction could assist the H atom transfer involved in the mechanism proposed to be behind the catalytic activity of related copper complexes [52].

Regarding the dipeptide complexes, the experimental evidence indicated that the redox activity was highly variable (Figure 4 and Table 2). The three members modeled by DFT (Cu–AlaPhe, Cu–AlaGly and Cu–PheAla) displayed medium dipole moments and copper atomic charges. Therefore, the electrostatic interactions with the O_2_^−^ anion were less important, and the electronic factors started to take over. In fact, the best SOD mimic, Cu–AlaPhe, was the only complex that had the β LUMO centered in the copper ion (Cu contribution: 68.8%; Figure 5a), while keeping a high local electrophilicity and softness at the metal center. Moreover, the O_2_^−^ ligand was further stabilized by establishing attractive anion-π interactions with the phenyl substituent of the phenylalanine fragment (Figure 5f). Strikingly, for the isomer Cu–PheAla, where the phenyl ring is located further away from the copper ion, the scenario changed substantially. It did not establish the same O_2_^−^–π interaction and the LUMO was shifted towards the carbonyl group. In this regard, the computational model suggests that the particular position of the phenyl ring in Cu–AlaPhe promotes the formation of a highly reactive complex (IC_50_ = 5.0 μM), which has (i) a metal center that is more electrophilic and softer, (ii) an adequate location for the LUMO and (iii) stabilizing interactions with the superoxide anion.

In contrast, the homoleptic Cu–AA compounds proved to be moderate SOD mimics, with activities that were almost independent of the complex chemical nature (Table 2). According to the computational results, Cu–Ala displayed the lowest dipole moment and copper atomic charge. Therefore, the electrostatic interactions were not expected to be a significant contribution during the reaction with the superoxide anion. Besides, the electronic descriptors indicated that its local electrophilicity, softness and Cu contribution to LUMO were intermediate, and no specific attractive interactions were set up with the O_2_^−^ fragment (Figure 5h). All this accounts for its medium antioxidant ability (IC_50_ = 32.3 μM).

#### 2.6.2. Pro-Oxidant Activity

All the complexes tested produced OH from H_2_O_2_, probably via the formation of an adduct with H_2_O_2_ and the subsequent formation of the intermediate Cu(ligands)-OOH^−^ upon deprotonation (see Figure 6) [53]. However, the homoleptic complexes with amino acids displayed the highest pro-oxidant activity. In contrast, the heteroleptic Cu–dipeptide–phen complexes were the least active. In the middle, the OH production of the Cu–dipeptide complexes showed substantial variability. The calculated reactivity indexes associated with the charge distribution (μ, copper atomic charge), shown in Table 3, did not follow any clear trend, indicating that the electrostatic interactions with the neutral molecule H_2_O_2_ did not play an important role in determining the pro-oxidant activity. Nevertheless, some electronic features can be used to rationalize the general tendency (Table 4). Among the complexes that exhibited the highest pro-oxidant ability (Cu–Ala, Cu–AlaGly and Cu–AlaGly–phen), the activities increased with the global hardness and copper electrophilicity. The rest of the complexes (Cu–AlaPhe and Cu–PheAla) displayed the highest condensed local softness levels at the copper ion, making the metal center less hard. This possibly increased the activation barrier of the reaction with H_2_O_2_ (a harder substrate than the superoxide anion).

Concerning the structural aspects of the adducts with H_2_O_2_ and OOH^−^, some interesting aspects arose (Figure 6). The most active complexes, Cu–Ala and Cu–AlaGly, formed adducts with hydrogen peroxide that had the H_2_O_2_ molecule near the metal center. This was possible due to the fact that the coordination environment lacked bulky groups, making the axial labile coordination sites freely accessible. The steric hindrance of the coordination polyhedral has been associated with low catalytic activities in other copper complexes [54]. For the rest of the complexes, the phen coligands or the phenyl substituent resulted in the H_2_O_2_ molecule being located further away from the Cu(II) ion. Upon protonation, the flexibility of the coordination polyehdra seems to have been a crucial factor in stabilizing the Cu(ligands)-OOH^−^ species. The homoleptic complexes with amino acids can twist the coordination scheme, allowing the OOH^−^ species to remain pentacoordinated (see the structure for Cu–Ala in Figure 6f). This fifth coordination bond stabilizes the intermediate.

For the Cu–dipeptide compounds, however, the coordinated water molecule was exchanged by the OOH^−^ ligand, giving rise to tetracoordinated complexes (Figure 6g,i,j). The rigidity of the coordination polyhedral brought about by the tridentate dipeptide ligand prevented the formation of a more stable pentacoordinated intermediate.

Lastly, the phen-containing heteroleptic complexes deserve a special comment. During the geometry optimization runs of [Cu(AlaGly)(OOH)phen] species, the phen ligand was displaced by the OOH^−^ anion to give a tetragonal coordination polyhedron (Figure 6h), similar to that found for the homoleptic dipeptide complexes (Figure 6g,i,j). This outcome was indicative of the fact that the phen coligand would hamper the formation of the intermediates involved in the ROS production from H_2_O_2_, which would have to be displaced from the coordination sphere, thus destabilizing the system. This could account, at least partially, for the very low level of OH production in the heteroleptic copper complexes.

## 3. Materials and Methods

### 3.1. Reagents

Analytical-grade chemicals and solvents needed for the complex synthesis were purchased from SIGMA and used as commercially available without further purification.

### 3.2. Synthesis of Complexes

#### 3.2.1. Homoleptic Copper Complexes with Amino Acids

The copper complexes with amino acids were obtained following well-known synthetic procedures [45,55].

In the case of the complexes with Gly, Ala and Ser, CuCO_3_Cu(OH)_2_ was used, while in the case of the complexes with Val and Ile, an aqueous solution with CuSO_4_·H_2_O was added.

The analytical results were:

For [Cu(Gly)_2_]·H_2_O, blue crystals, (FW: 229.64), yield: 46%, Anal. Calcd; found (%) C 20.9, N 12.19, H 4.35; C 21.28, N 12.15, H 4.32; λ_max_ (nm)/ε_M_ (M^−1^cm^−1^)(water) = 632/44.5.

For [Cu(Ala)_2_], blue-violet crystals, (FW: 328.77), yield: 65%, Anal. Calcd; found (%) C 30.04, N 11.68, H 5.01; C 30.43, N 11.87, H 5.12; λ_max_ (nm)/ε_M_ (M^−1^cm^−1^)(water) = 618/54.6.

For [Cu(Ser)_2_], blue powder, (FW: 271.68), yield: 60%, Anal. Calcd; found (%) C 26.31, N 10.23, H 4.38; C 26.81, N 9.63, H 4.59; λ_max_ (nm)/ε_M_ (M^−1^cm^−1^)(water) = 636/42.

For [Cu(Val)_2_], blue crystals, (FW: 295.80), yield: 30%, Anal. Calcd; found (%) C 40.57, N 9.46, H 6.76; C 40.81, N 9.60, H 6.75; λ_max_ (nm)/ε_M_ (M^−1^cm^−1^)(water) = 612/53.3.

For [Cu(Ile)_2_]·H_2_O, blue crystals, (FW: 341.84), yield: 93%, Anal. Calcd; found (%) C 42.12, N 8.19, H 7.60; C 42.64, N 8.36, H 7.88; λ_max_ (nm)/ε_M_ (M^−1^cm^−1^)(water) = 618/53.3.

#### 3.2.2. Homoleptic Copper Complexes with Dipeptides

Copper(II) dipeptide complexes were obtained according to the procedure previously reported [30]. In order to obtain adequate monocrystals, several tests were performed. One test involved the addition of an aqueous solution of NaPF_6_ over the aqueous solution of the complex. Only the complex Cu–AlaLeu presented PF_6_^−^ as counterion.

The analytical results were:

For [Cu(GlyVal)]·1/2H_2_O, blue crystals, (FW: 244.7), yield: 80%, Anal. Calcd; found (%) C 34.35, N 11.45, H 4.35; C 34.52, N 11.51, H 5.53; λ_max_ (nm)/ε_M_ (M^−1^cm^−1^)(water) = 635/80. 

For [Cu(ValGly)], blue crystals, (FW: 235.73), yield: 80%, Anal. Calcd; found (%) C 35.67, N 11.88, H 5.13; C 35.92, N 11.96, H 5.58; λ_max_ (nm)/ε_M_ (M^−1^cm^−1^)(water) = 636/68. 

For [Cu(AlaGly)])]·H_2_O, blue sheets, (FW: 226.7), yield: 70%, Anal. Calcd; found (%) C 26.45, N 12.36, H 4.90; C 26.59, N 12.23, H 5.12; λ_max_ (nm)/ε_M_ (M^−1^cm^−1^)(water) = 635/65. 

For [Cu(AlaPhe)])], blue crystals, (FW: 297.5), yield: 80%, Anal. Calcd; found (%) C 48.35, N 9.40, H 4.70; C 48.15, N 9.30, H 4.81; λ_max_ (nm)/ε_M_ (M^−1^cm^−1^)(water) = 625/60. 

For [Cu(PheAla)])]·1/2H_2_O, blue crystals, (FW: 306.8), yield: 60%, Anal. Calcd; found (%) C 46.93, N 9.13, H 4.89; C 47.12, N 9.19, H 4.23; λ_max_ (nm)/ε_M_ (M^−1^cm^−1^)(water) = 634/86. 

For [Cu(AlaLeu)_3_(H_2_O)(CO_3_)]·PF_6_·H_2_O, blue crystals, (FW: 1065.35), yield: 70%, Anal. Calcd; found (%) C 31.40, N 7.85, H 5.54; C 32.23, N 8.17, H 6.09; λ_max_ (nm)/ε_M_ (M^−1^cm^−1^)(water) = 626/70. 

#### 3.2.3. Heteroleptic Copper Complexes with Peptides and 1,10 Phenantroline

The heteroleptic copper complexes were synthesized according to the procedure previously reported [26].

The analytical results were:

For [Cu(AlaGly)phen])]·5H_2_O, blue crystals, (FW: 550.06), yield: 60%, Anal. Calcd; found (%) C 42.72, N11.72, H 5.48; C 42.54, N 11.73, H 5.74; λ_max_ (nm)/ε_M_ (M^−1^cm^−1^)(water) = 636, 850sh/99.

For [Cu(Phe-Ala)(phen)]·4H_2_O, blue-purple crystals, (FW: 549.98), yield: 60%, Anal. Calcd; found (%): C 52.40, N 10.19, H 5.50; C 52.11, N 10.22, H 5.68; λ_max_ (nm)/ε_M_ (M^−1^cm^−1^)(water) = 634, 850sh/96. 

For [Cu(PheVal)phen]·4.5H_2_O, Anal. Calcd; found (%) C 53.19, N 9.54, H 6.01; C 53.21, N 9.53, H 5.45; λ_max_ (nm)/ε_M_ (M^−1^cm^−1^)(water) = 630, 850sh/113.

### 3.3. Characterization

Light-atom elemental analysis of the coordination compound was performed on a Thermo Scientific Flash 2000 analyzer. FT-IR spectra were registered as solid solutions (1%) on KBr in the 4000 to 400 cm^−1^ range on a Shimadzu Prestige 21 spectrometer. Electronic spectra were measured on a Shimadzu UV 1603 spectrophotometer, using 1 cm path-length quartz cells. 

Single crystals of [Cu(AlaLeu)_3_(H_2_O)(CO_3_)]·PF_6_·H_2_O were obtained by slow evaporation. A suitable crystal was selected and measured using graphite monochromated MoKα radiation (0.71073 Å) at 120.0(2) K on a KAPPA-CCD diffractometer. Using Olex2 [56], the structure was solved with the SHELXT [57] structure solution program using intrinsic phasing and refined with the SHELXL refinement package using F^2^ least-squares minimization [58].

All non-hydrogen atoms were refined using anisotropic displacement parameters, whereas H atoms were geometrically position and refined isotropically using the *riding* model. 

The –CH-(CH_3_)_2_ side-groups of the alanine residues display varying degrees of disorder, as evidenced by their rather large anisotropic displacement factors and by the fact that some C-C distances tend to refine to unreasonably short values. In particular, the distances C37–C39 were refined with a 70/30 two-position model.

The asymmetric unit also includes four water molecules, three bonded to each of the Cu ions and one present as a crystallization solvent.

Structure visualization and image preparation was done using Mercury [59]. A summary of the crystallographic data, experimental details and refinement results are listed in Table 5. CIF files were deposited in the Cambridge Structural Database with Deposition Number 2113292. Copies are available free of charge through the access structures applet in the CCDC webpage.

Table 5 summarizes the crystal data and the refinement parameters for [Cu(AlaLeu)_3_(H_2_O)(CO_3_)]·PF_6_·H_2_O.

### 3.4. Oxidative Damage to 2-Deoxi-d-ribose (TBARS Method)

Technique: The reactions were performed with a pH 7.4 phosphate buffer containing 50 mM 2-deoxy-d-ribose, 3.00 mM H_2_O_2_ and 40–200 µM copper(II) complex at a final volume of 1 mL. The solution was incubated at 37.0 °C for 40 min, and then 1 mL of 1%(*w*/*v*) 2-thiobarbituric acid was added and the solutions incubated again at 90.0 °C for 15 min. After cooling, the absorbance of the solutions was measured at 532 nm. The pro-oxidant activity is expressed as MDA equivalents (μM) using a calibration curve. All experiments were performed in duplicate.

### 3.5. Determination of SOD-like Activity

Technique: Aqueous solutions of 3 mM Xanthine, 0.75 mM NBT, 3 mM Na_4_EDTA, 1.5 mg/mL bovine albumin, phosphate buffer pH = 7, 10 mg/mL xanthine oxidase, 6 mM CuCl_2_ and the compound under study were prepared in a range of concentrations of 1 × 10^−3^–1 × 10^−7^ M.

In each reaction tube, 0.2 mL of xanthine solution, 0.1 mL of NBT solution, 0.1 mL of albumin solution, 1mL of the corresponding dilution of the study compound and 1.8 mL of phosphate buffer were added. Each tube was incubated at 25 °C and 0.1 mL of xanthine oxidase was added. It was allowed to react at 28 °C and the reaction was stopped with CuCl_2_. The absorbance of each tube was measured at 560 nm.

The corresponding blanks were made without the complex and contained only the complex, and it was verified that the complex did not affect the activity of xanthine oxidase. 

For comparative purposes, the activity of native superoxide dismutase from bovine erythrocytes and of the CuSO_4_·5H_2_O was also considered. 

### 3.6. DFT Calculations

Initial geometries for the Cu(II) complexes were built taking into account the crystallographic data [36,60] and the structural information in solution derived from the electronic spectra, completing the coordination sites with water molecules when necessary. For the complex species containing H_2_O_2_, OOH^−^ or O_2_^−^, the input geometries were constructed with the oxygenated ligand initially coordinated along the direction indicated by the electric dipole moment vector (μ) and the electrostatic potential. The initial structures were optimized in aqueous solution by means of density functional theory (DFT) [61,62], as implemented in Gaussian 09 [63]. The calculations were run at the B3LYP/6-31 + G(d,p) level of theory, using an ultrafine integration grid and under implicit solvation simulated by an SMD solvation model [64]. For the superoxide-containing systems, both possible spin states (singlet and triplet) were tested. After the optimization runs, the water molecules not coordinated to the Cu(II) ion were removed and the structures were re-optimized under the same conditions. All the final optimum geometries corresponded to energetic minima, with the nature of the stationary points verified by a frequency analysis. 

Reactivity descriptors within the conceptual density functional theory [49] were calculated for the DFT-optimized structures, employing the Hirshfeld charges (dipole-corrected) and partitions with the program Multiwfn (version 3.7) [65]. The weak interactions were characterized using the noncovalent interaction (NCI) method [50]. The results were rendered with Gaussview 6.0 [66] and VMD 1.9.3. [67].

## 4. Conclusions

In this work, the first crystal structure of [Cu_3_(AlaLeu)_3_(H_2_O)_3_(CO_3_)]·PF_6_·H_2_O was presented and discussed. The dipeptide coordinated in a similar way to other dipeptide complexes, acting as a tridentate ligand through the terminal amino and carboxylate groups, in addition to the amidic nitrogen atom. Remarkably, it displayed a supramolecular structure significantly different from those previously obtained under the same synthetic and crystallization protocols, as it was the first homotrinuclear carbonato-bridged copper(II) complex with a dipeptide moiety.

On the other hand, the redox behaviors of a series of homoleptic copper(II) complexes with amino acids and dipeptides, as well as heteroleptic complexes containing both dipeptides and 1,10-phenanthroline, were characterized through the evaluation of the pro-oxidant (TBARS; OH production) and antioxidant (SOD-like activity) activities. A comparative analysis of the results showed that the Cu–AA complexes were high ROS producers and moderate SOD mimics, while the Cu–dipeptide–phen complexes were good SOD mimics and poor ROS producers. Meanwhile, the activity of Cu–dipeptide complexes was strongly dependent on the ligand.

The DFT computational analysis showed that the SOD-like ability was modulated by the electrostatic interactions with the superoxide anion, which increased with the positive charge at the metal center and the magnitude of the dipole moment. The inclusion of phen as coligand proved to be an efficient way to enhance the electrostatic interactions, as well as to stabilize the superoxide-complex intermediate, enhancing the antioxidant activity. Another way to increase the SOD-like activity was to position a phenyl ring near the metal center (as in Cu-AlaPhe), since it led to a higher contribution of the copper ion to the LUMO, in conjunction with greater condensed local softness and electrophilicity in the metal center. 

Concerning the pro-oxidant ability, the computational evidence indicated that the OH production from H_2_O_2_ was not modulated by electrostatic interactions. Rather, a high level of ROS production was in fact associated with higher global hardness and copper electrophilicity, along with lower condensed-to-copper local softness. Another important feature that had a profound impact on the pro-oxidant ability of the copper complexes was the nature of the coordination environment. When it lacked bulky groups, it allowed the H_2_O_2_ to easily reach the labile coordination sites, thermodynamically favoring the reaction. This is why the phen coligand or the phenyl substituent in the dipeptide ligands precluded the approach of the H_2_O_2_ molecule, hampering the OH production. Finally, the flexibility of the coordination polyhedra proved to be a crucial factor in stabilizing the Cu(ligands)-OOH^−^ species, giving rise to better pro-oxidant profiles. These findings reveal, for the first time, the structural and electronic bases behind the high ROS production displayed by homoleptic complexes with amino acids, for which the flexibility of the coordination sphere and the absence of bulky ligand substituents make them the best pro-oxidant candidates.

## Figures and Tables

**Table 1:** Coordinative bond distances.

Bond	Distance (Å)	Bond	Distance (Å)	Bond	Distance (Å)
Cu1–N11	2.014(9)	Cu2–N21	2.040(10)	Cu3–N31	2.011(10)
Cu1–N12	1.903(10)	Cu2–N22	1.891(10)	Cu3–N32	1.897(9)
Cu1–O1	1.956(8)	Cu2–O2	1.954(8)	Cu3–O3	1.923(8)
Cu1–O12	1.996(7)	Cu2–O22	2.005(8)	Cu3–O32	2.008(8)
Cu1–O1W	2.499(9)	Cu2–O2W	2.547(11)	Cu3–O3W	2.407(9)

**Table 2:** Concentration required to yield 50% inhibition of the NBT reduction (IC_50_). Boxplots of the IC_50_ for each group of complexes are also included.

Type	Complex	IC_50_ (μM)
Cu–AA	Cu–Val	30.3 [44]
	Cu–Ala	32.3 [44]
	Cu–Ile	33.1 [44]
	Cu–Gly	34.0 [44]
	Cu–Ser	37.2 [44]
Cu–dip	Cu–AlaPhe	5.0 [36]
	Cu–AlaLeu	22 [45]
	Cu–PheAla	45
	Cu–GlyVal	56
	Cu–ValGly	57
	Cu–AlaGly	124 [45]
Cu–dip–phen	Cu–AlaGly–phen	10
	Cu–PheAla–phen	2.5
	Cu–PheVal–phen	3.7
	[Cu(H_2_O)_6_]^2+^	30 [46]
	Cu–SOD *	0.04

* Native superoxide dismutase from bovine erythrocyte.

**Table 3:** Reactivity descriptors. The complexes are ordered from high to low in terms of SOD-like activity.

Complex	Global Descriptors	Condensed Descriptors (Cu Atom)			Cu Contribution (%)
	Dipole Moment (D)	Hirshfeld Atomic Charge	Electrophilicity	Local Softness ^a^	β LUMO
Cu–AlaPhe	19.8	1.15	1.74	5.27	68.8
Cu–AlaGly–phen	26.1	1.31	1.05	3.24	0.37
Cu–Ala	0.46	0.98	1.37	3.79	1.87
Cu–PheAla	19.1	1.17	1.80	5.39	0.54
Cu–AlaGly	20.2	1.00	1.35	3.96	0.85

^a^ *s*^+^_Cu_ = (VIP − VEA)^−1^ *f*^+^_Cu_, where VIP = vertical ionization potential (*E*(N − 1) − *E*(N)), VEA = vertical electron affinity (*E*(N) − *E*(N + 1)), and *f*^+^_Cu_ is the condensed-to-copper Fukui function for the nucleophilic attack.

**Table 4:** Reactivity descriptors. The complexes are ordered from high to low in terms of OH production.

Complex	Global Descriptors	Condensed Descriptors (Cu Atom)	
	Hardness η (eV)	Electrophilicity	Local Softness ^a^
Cu–Ala	3.65	1.37	3.79
Cu–AlaGly	3.53	1.35	3.96
Cu–AlaGly–phen	3.37	1.05	3.24
Cu–AlaPhe	3.50	1.74	5.27
Cu–PheAla	3.48	1.80	5.39

^a^ *s*^+^_Cu_ = (VIP − VEA)^−1^ *f*^+^_Cu_, where VIP = vertical ionization potential (*E*(N − 1) − *E*(N)), VEA = vertical electron affinity (*E*(N) − *E*(N + 1)), and *f*^+^_Cu_ is the condensed-to-copper Fukui function for the nucleophilic attack.

**Table 5:** Summary of crystal data and structure refinement parameters for [Cu(AlaLeu)_3_(H_2_O)(CO_3_)]·PF_6_·H_2_O.

Empirical Formula	C_28_ H_53_ Cu_3_ F_6_ N_6_ O_16_ P
Formula weight	1065.35
Temperature	120(2) K
Wavelength	0.71073 Å
Crystal system	Orthorhombic
Space group	P2_1_2_1_2_1_
Unit cell dimensions	*a* = 10.420(1) Å
	*b* = 16.295(1) Å
	*c* = 28.236(1) Å
Volume	4794.3(6) Å^3^
Z	4
Density (calculated)	1.476 Mg/m^3^
Absorption coefficient	1.439 mm^−1^
F(000)	2188
Crystal size	0.22 × 0.21 × 0.18 mm^3^
Theta range for data collection	2.32 to 25.00°.
Index ranges	−12 ≤ *h* ≤ 12, −19 ≤ *k* ≤ 19, −33 ≤ *l* ≤ 33
Reflections collected	8081
Independent reflections	8081 (R(int) = 0.0560)
Completeness to theta = 25.00°	99.2%
Absorption correction	Semi-empirical from equivalents
Max. and min. transmission	0.7817 and 0.7425
Refinement method	Full-matrix least-squares on F^2^
Data/restraints/parameters	8081/2/541
Goodness-of-fit on F^2^	1.044
Final R indices (I > 2σ (I))	R1 = 0.0546, wR2 = 0.1330
R indices (all data)	R1 = 0.0663, wR2 = 0.1409
Absolute structure parameter	0.018(16)
Largest diff. peak and hole	1.224 and −0.560 e.Å^−3^

## Data Availability

Not applicable.

## Supplementary Materials

The following are available online. Table S1. Cambridge Structural Database Refcodes and counterions. Figure S1. DFT-optimized geometries in aqueous solution of the complexes formed by superoxide with Cu-AlaPhe (a), Cu-AlaGly-phen (b), Cu-Ala (c), Cu-PheAla (d) and Cu-AlaGly (e). Figure S2. DFT-optimized geometries in aqueous solution of the adducts formed by hydrogen peroxide with Cu-Ala (a), Cu-AlaGly (b), Cu-AlaGly-phen (c), Cu-AlaPhe (d) and Cu-PheAla (e).

## Sample Availability

Samples of the compounds are available from the authors.
