# Supplementary material for: Comparative Study of Antioxidant and Pro-Oxidant Properties of Homoleptic and Heteroleptic Copper Complexes with Amino Acids, Dipeptides and 1,10-Phenanthroline: The Quest for Antitumor Compounds
Source: Molecules. 2021 Oct 28;26(21):6520. doi: 10.3390/molecules26216520 (PMC8587672; doi:10.3390/molecules26216520)
Supplement: Supplementary file 1 [file molecules-26-06520-s001.zip › molecules-1433163-supplementary.pdf]

Supplementary materials

**Table S1. Cambridge Structural Database Refcodes and counterions**

| Refcode | Anion |
|---|---|
| ANAXAQ | $PF_6^-$ |
| AYEBIQ | $F_3C\text{-}SO_3^-$ |
| AYEBOW | $F_3C\text{-}SO_3^-$ |
| CEKXOH | $ClO_4^-$ |
| CEKXUN | $ClO_4^-$ |
| CEKYAU | $PF_6^-$ |
| COPYCU | $NO_3^-$ |
| FUNTEQ | $BF_4^-$ |
| GAJHUX | $F_3C\text{-}SO_3^-$ |
| GIMHIW | $ClO_4^-$ |
| GUBLOG | $ClO_4^-$ |
| HISSAG | $PF_6^-$ |
| HISSAG01 | $PF_6^-$ |
| IBOLAO | $ClO_4^-$ |
| IBOLES | $NO_3^-$ |
| IFIDOR | $ClO_4^-$ |
| IHILAP | $ClO_4^-$ |
| IMAKAK | $PF_6^-$ |
| IMIHOB | $ClO_4^-$ |
| IPUKAF | $ClO_4^-$ |
| MOGPUT | $BF_4^-$ |
| MUWWOQ | $ClO_4^-$ |
| NOJZOB | $ClO_4^-$ |
| OGEHIR | $ClO_4^-$ |
| QOFKEB | $ClO_4^-$ |
| RAMVIL | $ClO_4^-$ |
| TAMMAW | $ClO_4^-$ |
| TIBYIP | bis(closododecaborate) |
| TUDBIG | 2-methylpropane-2-sulfonate |
| TUDBOM | 2-methylpropane-2-sulfonate |
| WOJSOD | $BF_4^-$ |
| ZINXAV | $ClO_4^-$ |
| ZINXAV10 | $ClO_4^-$ |
| ZOKDIM | $ClO_4^-$ |
| ZOKDIM01 | $ClO_4^-$ |

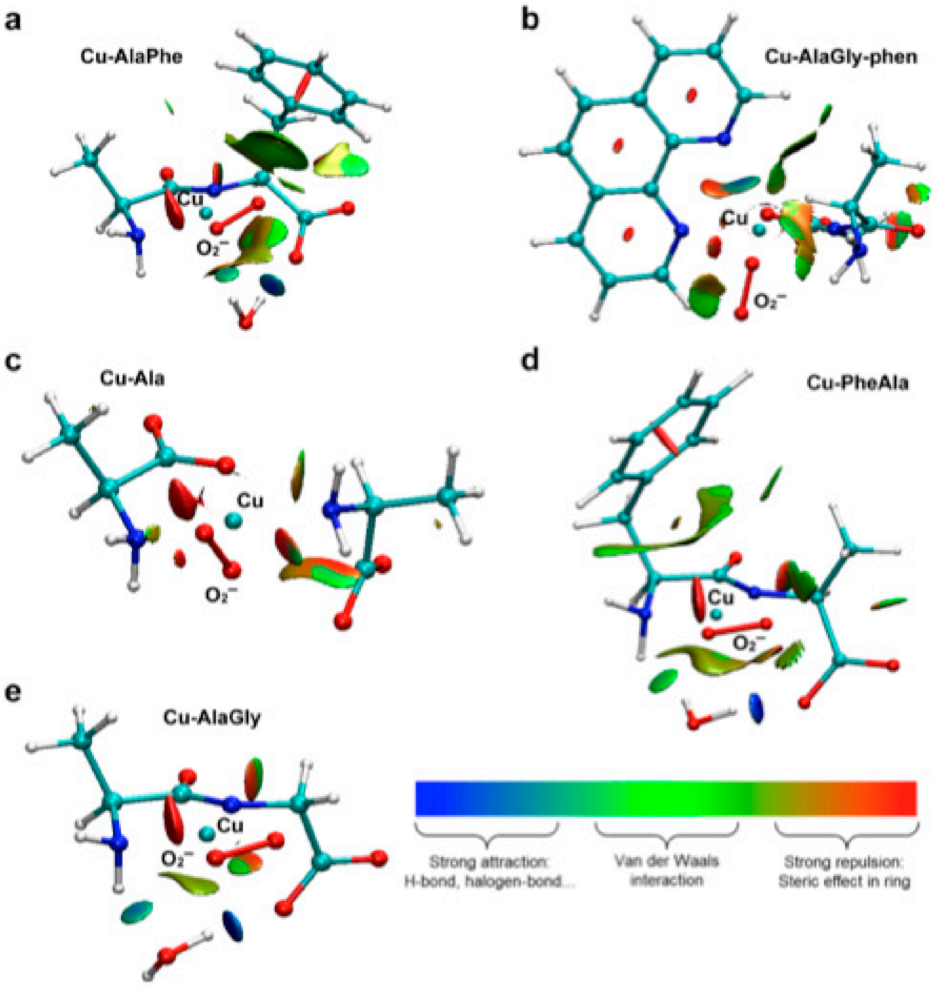


Figure S1. DFT-optimized geometries in aqueous solution of the complexes formed by superoxide with Cu-AlaPhe (a), Cu-AlaGly-phen (b), Cu-Ala (c), Cu-PheAla (d) and Cu-AlaGly (e). A visual representation of the weak interactions using the noncovalent interaction (NCI) method is also shown. The values of the product *sign*($\lambda_2$)ρ are represented with different colors and mapped on a reduced density gradient (RDG) isosurface (isovalue = 0.5), revealing the different weak interactions involved: H-bonds (blue), Van der Waals (green) and steric repulsion (red). $\lambda_2$ = second largest eigenvalue of the Hessian matrix of electron density. ρ = electron density.

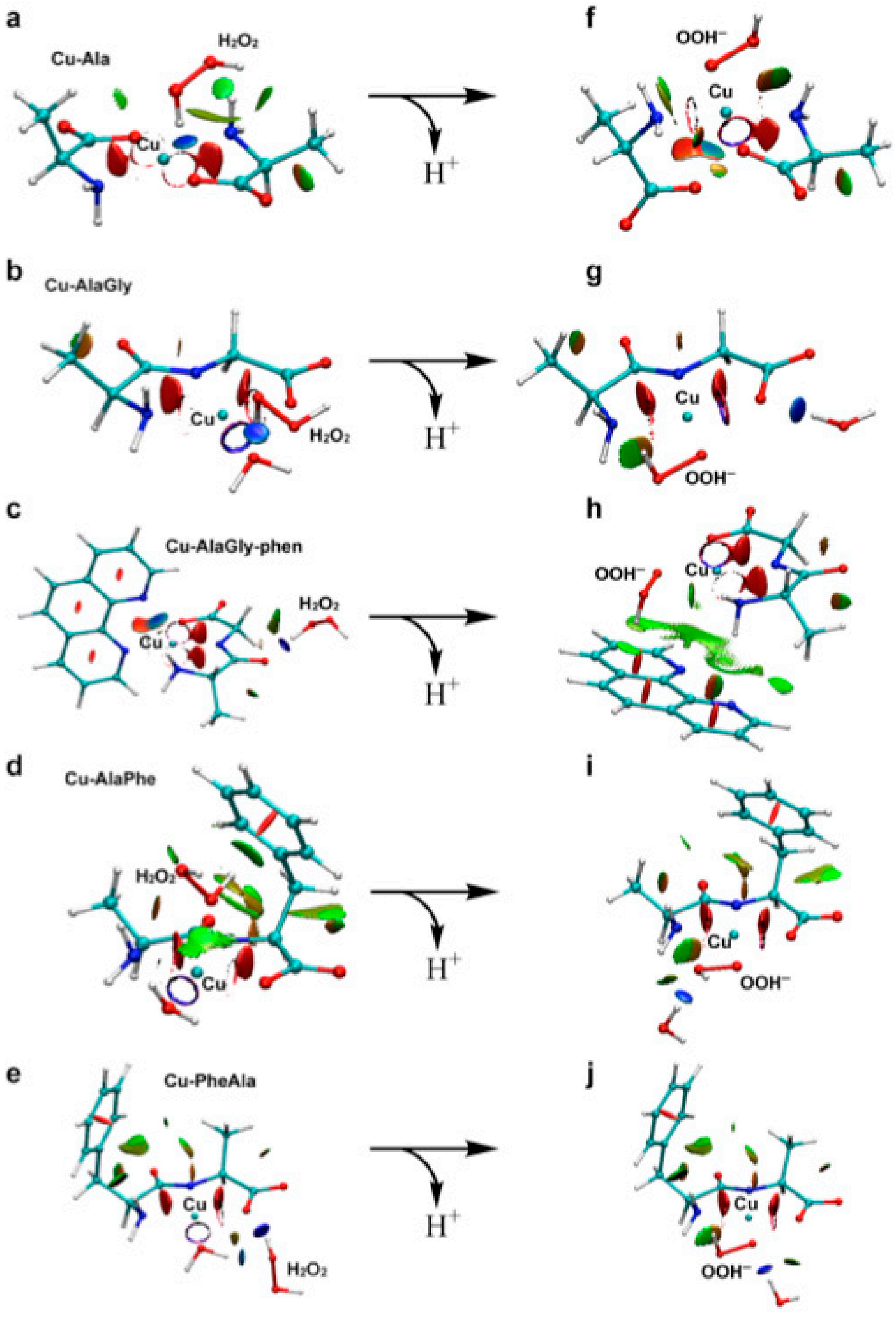


Figure S2. DFT-optimized geometries in aqueous solution of the adducts formed by hydrogen peroxide with Cu-Ala (a), Cu-AlaGly (b), Cu-AlaGly-phen (c), Cu-AlaPhe (d) and Cu-PheAla (e). In (f) – (j), the optimized structures of the corresponding $OOH^-$ complexes are depicted. A visual representation of the weak interactions using the noncovalent interaction (NCI) method is also shown. The values of the product *sign*($\lambda_2$)ρ are represented with different colors and mapped on a reduced density gradient (RDG) isosurface (isovalue = 0.5), revealing the different weak interactions involved: H-bonds (blue), Van der Waals (green) and steric repulsion (red). $\lambda_2$ = second largest eigenvalue of the Hessian matrix of electron density. ρ = electron density.
